# Optical properties of fully conjugated cyclo[*n*]thiophenes – An experimental and theoretical approach

**DOI:** 10.3762/bjnano.2.78

**Published:** 2011-10-25

**Authors:** Elena Mena-Osteritz, Fan Zhang, Günther Götz, Peter Reineker, Peter Bäuerle

**Affiliations:** 1 Institute of Organic Chemistry II and Advanced Materials, University of Ulm, Albert-Einstein-Allee 11, 89081 Ulm, Germany; 2 Institute of Theoretical Physics, University of Ulm, Albert-Einstein-Allee 11, 89081 Ulm, Germany

**Keywords:** conjugated macrocycles, Frenkel exciton model, oligothiophene, photophysical properties

## Abstract

Optical properties of two series of fully conjugated cyclo[*n*]thiophenes were analyzed experimentally and theoretically. The absorption spectra reveal a shift to higher wavelengths with increasing size of the cycles, which can be successfully described by an excitonic approach based on a Frenkel exciton Hamiltonian. Furthermore, intriguing new bands in the absorption and fluorescence spectra of the smaller macrocycles disclose the dominance of their ring strain.

## Introduction

In the last few decades organic conjugated polymers and oligomers, in particular poly- and oligothiophenes, have attracted a broad interest due to their excellent electronic and transport properties in the solid state, which allow their application in a variety of organic-electronic devices, such as organic field-effect transistors, organic solar cells, and sensors [1–4]. Typically, such π-conjugated systems comprise extended linear one-dimensional (1D) structures showing interesting optoelectronic properties. In the solid state they represent organic semiconductors, whereas by doping with oxidants metallic states with higher conductivities can be achieved. For various series of 1D linear oligothiophenes, which, in contrast to their polydisperse polymeric counterparts, exhibit defined molecular structures, it was proven that the physical properties correlate well with the length of the conjugated chain [5–8]. However, in particular for the shorter oligomers, end-effects imposed by the end groups perturb structure–property correlations, whereas for longer derivatives saturation of, e.g., optical transitions occurs leading to a limiting value. In this respect, 2D macrocyclic systems, cyclo[*n*]thiophenes (C*n*T) [9–11], which are shape-persistent and cyclically conjugated, were recently introduced by our group. They are not only theoretically most-interesting systems [12–16] providing an infinite π-conjugated chain like an idealized polymer, but they also represent a novel class of organic semiconductors without end-effects exhibiting fascinating optical [17–20] and self-assembling properties [21–23].

In a statistical macrocyclization approach under high-dilution conditions, the first cyclic representatives **C12T**, **C16T**, and **C18T** were prepared starting from terminally ethynylated terthiophenes, in only low yields and quantities, which is a general observation for the statistical synthesis of macrocycles [24–26]. Somewhat later, we developed a novel method using the same ethynylated oligothiophenes and Pt(II)-precursors as templates leading to stable coordinatively bound metallomacrocycles, which were transformed to the corresponding diacetylene-bridged macrocycles by elimination of the metal centers and simultaneous C–C bond formation. In a final step, the diacetylene units were subsequently transformed to thiophene units forming the final cyclothiophene. By this “metal**–**template approach”, among other derivatives the series was extended to **C8T** as the smallest member and the overall yield was improved to around 10% [6–7]. More recently, by applying Pt(II)-oligothienyl complexes [27], a more direct general and highly effective “one-pot” synthesis of cyclo[*n*]thiophenes was developed. By using linear pentameric quinquethiophene **L5T** as a building block, a series of individual macrocycles C*n*T, from **C10T** to an unprecedented size up to **C35T**, was obtained in an excellent overall yield of around 60%. For the first time, C*n*Ts including members with an odd number of repeating units became available on a preparative scale. Thus, the following macrocycles were isolated and characterized: **C10T**, **C15T**, **C20T**, **C25T**, **C30T**, and **C35T** [28]. X-ray structural analysis of **C10T** and characterization of its charged states revealed an unusual polaron pair structure of doubly oxidized **C10T^2+^** serving as a model for charged and conducting states in linear oligo- and polythiophenes [29].

In this article, we present and analyze optical data on two larger series of cyclo[*n*]thiophenes C*n*T, series I and series II, which differ in ring size and substitution pattern of the solubilizing butyl side chains. The obtained structure–property relationships were further analyzed by our theoretical model based on Frenkel exciton theory. The comparison of the experimental with the theoretical spectral parameters gave valuable insights into the electronic structure, because they correlate the monomer transition energy (ω_0_), the magnitude of the electronic coupling between the thiophene monomers in the macromolecules (J), and the extent of the delocalized π-conjugated system [17].

## Experimental

The solutions were freshly prepared with chloroform (Merck, UVASOL). UV–vis absorbance spectra were recorded on a Perkin-Elmer Lambda 19 spectrometer, and corrected fluorescence spectra were recorded on a Perkin-Elmer LS 55 under ambient conditions.

## Results and Discussion

**Scheme 1:**
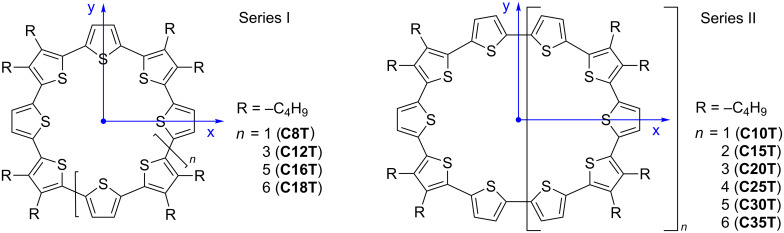
Chemical structure of the oligothiophene macrocycles (series I and II). The coordinate system used in the theoretical description is included.

The macrocycles examined in this study are depicted in Scheme 1.

The first family of compounds (series I) is represented by alternately substituted thiophene units, every second unit having two butyl side chains in the ß-positions leading to highly symmetrical derivatives: **C8T**, **C12T**, **C16T**, and **C18T**. Due to the pentameric starting material **L5T** with dibutyl substitution at thiophenes 2 and 4, in series II a different alkyl chain substitution pattern resulted and allowed synthesis of macrocycles composed of an even or odd number of thiophene rings. Therefore, a much more extended series from **C10T** (cyclodimer) as the smallest member to **C35T** (cycloheptamer) as the largest was obtained.

The photophysical properties of the two series of macrocycles were analyzed by absorption and fluorescence spectroscopy. The data are summarized in Table 1. Two linear oligothiophenes, **L5T** and **L10T**, are included in the table as references having an identical substitution pattern to the macrocycles in series II. **L5T** is the building block used for the cyclization reactions and **L10T** the linear dimer.

**Table 1:** Photophysical data of the cyclothiophenes belonging to series I and II in comparison to the linear reference compounds. Maxima at the absorption and emission wavelengths, λ_max_^abs^ and λ_max_^em^ (absolute maximum underlined), the extinction coefficient ε and the normalized extinction coefficient (ε/*N*_r_). **L5T** and **L10T** denote the linear homologues. Data of series II were taken from [28].

C*n*T series		λ_max_^abs^ [nm]([eV])^a^	ε [L·mol^−1^·cm^−1^]^a^	ε/*N*_r_ [L·mol^−1^·cm^−1^]^a^		λ_max_^em^ [nm]([eV])^a^
I	II			I	II	

**8**		396, ~500 (3.13, ~2.48)	51300	6413		435, 452 (2.85, 2.74) // 567, 602 (2.19, 2.06)

	**10**	417, ~500 (2.97, ~2.48)	86000		8600	~568, ~629, 685 (~2.18, ~1.97, 1.81)

**12**		392 (3.16)	55000	4583		~556, 593, ~660 (~2.23, 2.09, ~1.88)

	**15**	423 (2.93)	119000		7933	582, ~603 (2.13, ~2.06)

**16**		414 (3.00)	97000	6106		577, ~615 (2.15, ~2.02)

**18**		421 (2.95)	123000	6833		575, ~610 (2.16, ~2.03)

	**20**	434 (2.86)	130000		6500	572, ~603 (2.17, ~2.06)

	**25**	440 (2.82)	163000		6520	570, ~607 (2.18, ~2.04)

	**30**	444 (2.79)	183000		6100	568, 607 (2.18, 2.04)

	**35**	445 (2.79)	196000		5600	567, 604 (2.19, 2.06)

**L5T**		392 (3.16)	28000	5600		

**L10T**		435 (2.85)	51000	5100		553, ~587 (2.24, ~2.11)

^a^Solvent: Dichloromethane.

**Figure 1:**
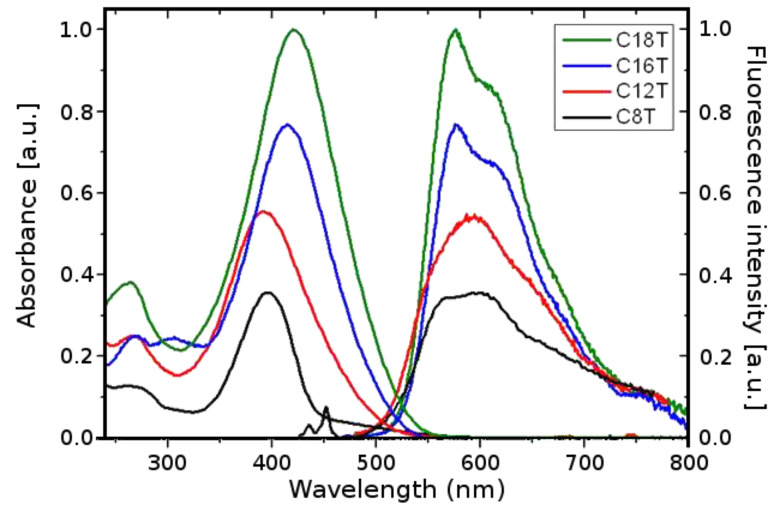
Absorption and fluorescence spectra of the macrocycles of Series I in dichloromethane (the excitation wavelength was chosen at the maximum of the absorption band).

**Figure 2:**
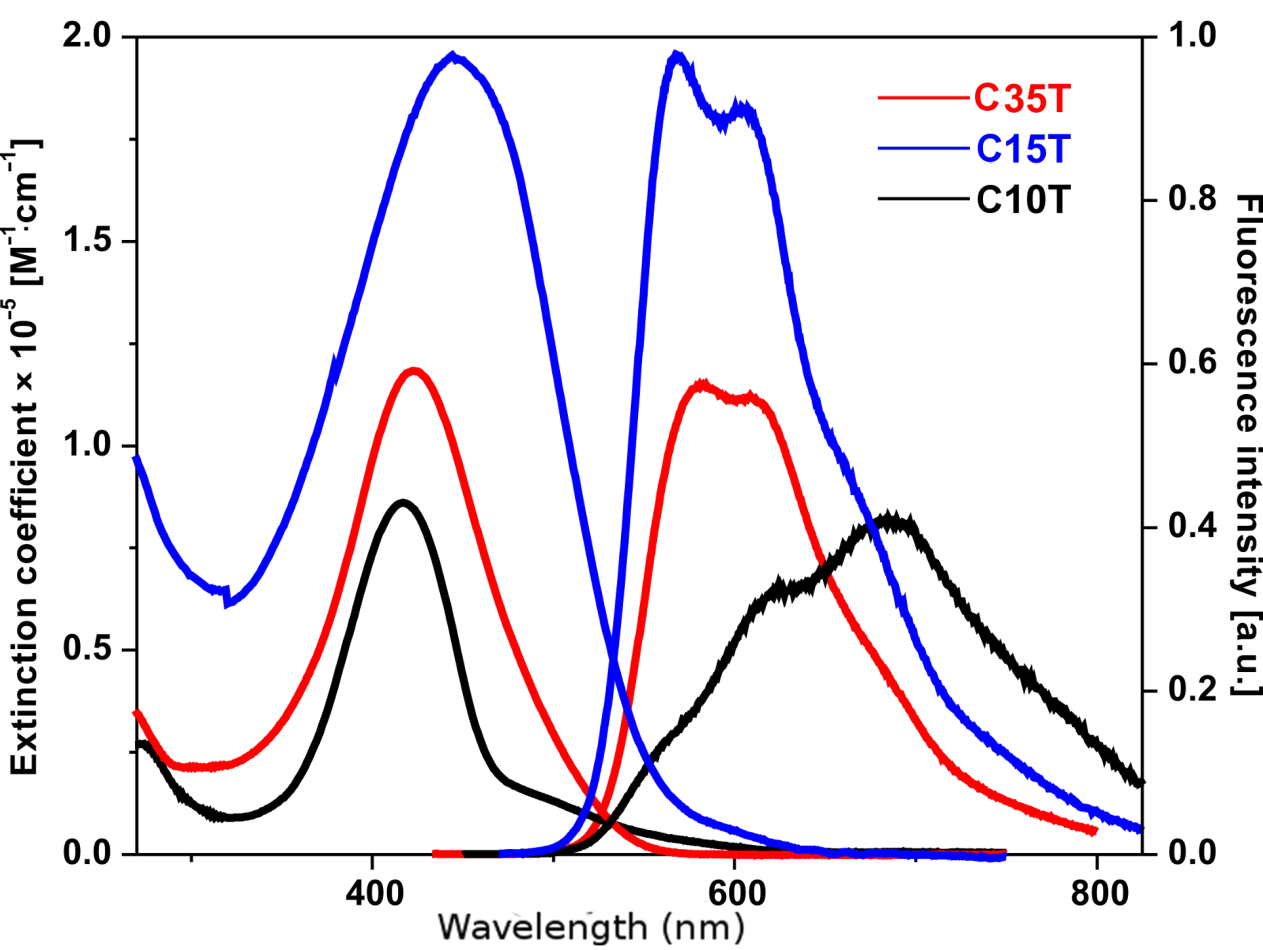
Representative absorption and fluorescence spectra of the smallest and largest macrocycles of series II in dichloromethane (taken from [28]). The excitation wavelength was chosen at the maximum of the absorption band.

Absorption spectra of the macrocycles in dichloromethane (DCM) at room temperature were characterized by several broad unstructured bands in the UV–vis region (Figure 1 and Figure 2).

In each series, the position of the low energy band, related to the π–π* transition of the macrocyclic systems, shifts on going from the smaller cycles to the larger ones. The absorption band (λ_max_ in Table 1) shifts to higher wavelengths with increasing number of thiophene units in the macrocycle (Δλ = 25 nm and Δλ = 28 nm in series I and II, respectively). The corresponding transition dipole lies in the molecular plane and is oriented almost parallel to the π-conjugated backbone, strongly dependent on the number of thiophene units composing the delocalized π-system, as in the case of the linear analogues [12]. Although a direct cross-correlation between the absorption of the two series seems inappropriate, the general trend shows that the macrocycles in series I absorb at higher energies than those of series II: **C15T** (λ_max_ = 423 nm) absorbs at a higher wavelength compared to the next higher macrocycle **C16T** (λ_max_ = 414 nm): The blue-shift of the absorption band in series I indicates slightly lower π-conjugation due to bigger distorsions of the thiophenes induced by steric interactions of the increased number of alkyl chains (8 versus 6 for **C16T** and **C15T**, respectively).

**Figure 3:**
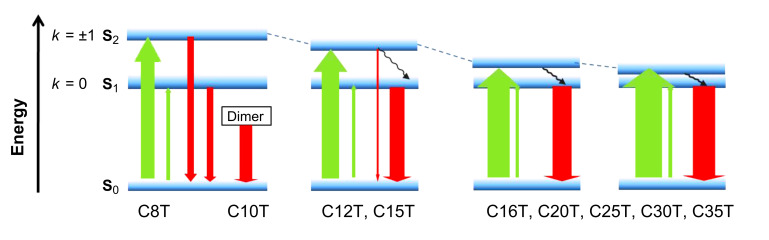
Sketch of the electronic transitions in the macrocycles: Ground state (S_0_), first (S_1_) and second (S_2_) excited state. Plain arrows are weighted by the transition probability. The black wavy arrows represent nonradiative deactivation.

A closer look at the smallest macrocycles (**C8T** and **C10T**) reveals a weak, but clear absorption band at lower energies (~500 nm), which in the case of the bigger macrocycles is overlapped and covered by the stronger main absorption band (Figure 1 and Figure 2). Because the selection rules for the electronic transitions of cyclic molecules substantially differ from those of the linear homologues, the transition to the lowest excited state in the macrocycles, which appears at ~2.48 eV independently of the ring size, is not allowed, whereas the degenerate second transition (corresponding to the intense absorption band) is permitted and ring-size dependent (see above) [12]. Figure 3 shows a sketch of the possible electronic transitions depending on the macrocycle size and taking into account the relative energies of the involved ground and excited states. The lack of vibronic structure in the absorption bands of all macrocycles indicates a nonplanar delocalized π-system with more or less well-pronounced torsion angles between the thiophene rings. This is in accordance with the behavior of the linear oligomers, which as well show a more pronounced aromatic structure in their ground state.

**Figure 4:**
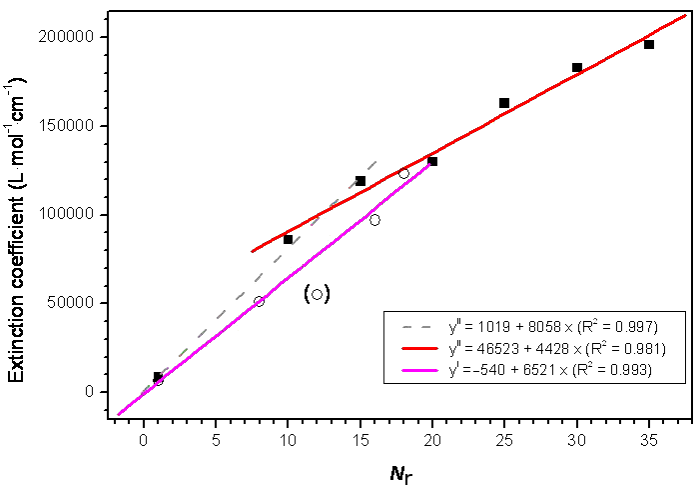
Extinction coefficient for the macrocycles of series I (circles) and II (squares) versus the number of thiophenes including the corresponding least-squares fit (y^I^ and y^II^) for the fit function ε = a + b*N*_r_. Compound **C12T** (circle in parentheses) was not included in the linear fit of series I.

In both series the extinction coefficient increases with increasing macrocyclic ring size, although the values for series I are smaller than for series II (Table 1 and Figure 4, also see below Equation 1 and Equation 2). This effect can be attributed to the already mentioned distinct substitution pattern for both series. The extinction coefficient versus number of thiophenes in the cycle for series II fits well to a linear trend (red curve in Figure 4) with a slope of 4428 L·mol^−1^·cm^−1^ per thiophene. This value is much lower than the normalized extinction coefficients (ε value/*N*_r_ the number of thiophenes in the macrocycle) in this series, which decay linearly with the macrocycle size (Table 1) reaching the normalized value for the linear oligomers **L5T** and **L10T** (~5100 L·mol^−1^·cm^−1^). Due to this discrepancy, a better fit can be calculated taking the last value as a supplementary point into account: A second linear fit can be calculated for the smaller macrocycles (up to **C15T**) with a slope of 8058 L·mol^−1^·cm^−1^. Interestingly, the normalized extinction coefficient remains almost constant in series I (exception **C12T**) with a value of about 6500 L·mol^−1^·cm^−1^.

The fluorescence spectra revealed a structured emission band, in which up to four vibronic contributions can be observed. In contrast to the discussed torsion of thiophene units in the ground state of the macrocycles, we assume that in the excited state the macrocyclic conjugated backbone tends to planarize reducing the torsional angles of the thiophene units and in accordance to the linear analogues tends to a more quinoidal structure.

The fluorescence spectra of the larger macrocycles are alike with respect to their shape and energy position with maxima at around 2.15 eV (series I) and 2.18 eV (series II). For the small macrocycles, however, striking differences concerning the emission-band shape emerge and new bands appear, pointing to a special behavior most probably related to the inherent ring strain in these smaller homologues. The most relevant features concern the appearance of a very weak fluorescence band in the case of **C8T** at ~450 nm and a more red-shifted emission in the case of **C10T**. The latter can be explained by the tendency of **C10T** to form dimers (excimers) in the excited state [29], which emit at lower energies with respect to the monomers (1.81 eV versus 2.18 eV). For the smallest cycle, **C8T**, we observe a weak structured emission band at much higher energy than the S_0_ ← S_1_ transition, whose origin can be attributed to the emission from the second excited state, S_2_ (Figure 1, Table 1 and Figure 3). This double fluorescence behavior becomes possible because an incomplete energy transfer to the S_1_ state occurs allowing partially the emission from the populated higher excited state S_2_.

In a previous theoretical work we showed that the optical excitations of thiophene-based linear and cyclic oligomers can be described by Frenkel excitons, which are delocalized over the oligomeric π-system [12,30]. Concerning the macrocycles of series I and II, we assume, as a first approach, that the electronic excitations of a thiophene subunit can be described by two relevant energy levels corresponding to the HOMO and the LUMO level. We assume that the energies of these states and the transition-matrix elements are essentially unmodified by the side chains. The Hamiltonian of the model then reads

[3]
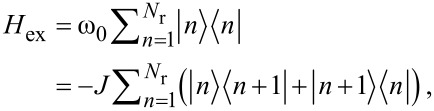


ω_0_ is the excitation energy, *N*_r_ the number of thiophene units in the macrocycle, *J* describes the transfer of the excitation energy between neighboring thiophenes and 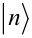 is the excited state at thiophene *n*. Throughout, we assume periodic boundary conditions, i.e., thiophenes 1 and *N*_r_ are nearest neighbors and complete the macrocyclic shape. The Schrödinger equation for this Hamiltonian can be represented as a difference equation. The energy eigenvalues and the eigenfunctions are given by the following expressions:

[4]
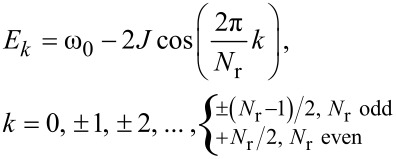


[5]
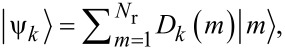


[6]
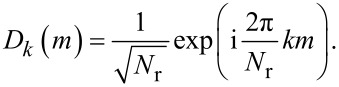


The allowed optical transitions between the ground state 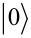 and the excited states (3) with energies given by (2) are determined by the matrix elements of the ring dipole operator:

[7]
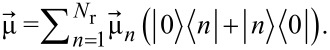


Here 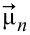 is the local optical dipole moment between the molecular ground and excited states. We assume that these dipole moments can have a component 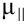 parallel to the z-axis and a component 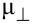 perpendicular to the z-axis, i.e., lying in the ring plane (x–y plane) and oriented in a tangential manner around the ring (Scheme 1) [31].

The evaluation of the z-component of the ring dipole moment 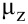 for the case that all molecular dipole moments 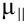 are equal results in the following selection rule:

[1]
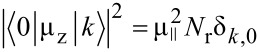


For the evaluation of the x-component of the ring dipole moment 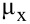 we again assume that the molecular dipole moments have the same 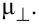 However, the in-plane component for each molecule is oriented parallel to the ring tangent and thus its orientation is shifted by 2π/*N*_r_ as compared to those of the neighboring molecules. The evaluation of the dipole moment operator gives in this case

[2]
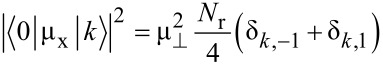


**Figure 5:**
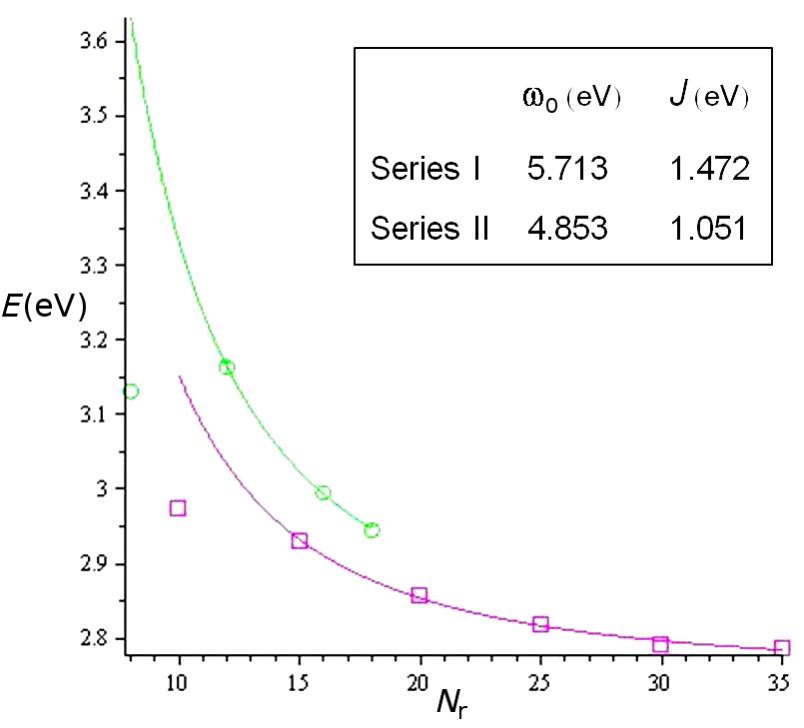
Diagram of the energy of the absorption band (eV) versus the number of thiophenes in the macrocycles of series I (circles) and II (squares), including the least-squares fit to Equation 4.

According to Equation 4 the two selection rules (Equation 1 and Equation 2) result in two different absorption lines, which agrees with our experimental finding of two absorption bands, more clearly observed for the small macrocycles. The first transition given for the component of the dipole moment perpendicular to the x–y plane has the selection rule *k* = 0 and their energy reads *E_k_* = ω_0_ – 2*J*, independent of the macrocycle size. The line position given for the component of the dipole moment in the x–y plane has the selection rule *k* = ±1 and the line position reads *E_k_* = ω_0_ – 2*J* cos(2π/*N*_r_). In the fit, using a least-squares procedure in the Maple software, we determine the parameters ω_0_ and *J*, related to the monomer transition energy (ω_0_) and the coupling energy between thiophene units (*J*). The results of the fit (in eV) are shown in Figure 5. The values corresponding to the smallest macrocycles in both series (**C8T** and **C10T**) were not included in the fitting, because of their evident deviation from the general trend. The reason for this behavior might be the ring strain of the macrocycle and the torsion that the individual thiophene rings experience due to their small ring size. This guess is supported by geometry calculations for **C8T** and X-ray structure analysis for **C10T** [10,29].

The macrocycles show an energetic decrease of their line positions, i.e., an increase of their wavelengths, with increasing ring size, which can be successfully described by Equation 4 and was observed experimentally. The circles show the experimental line positions of the macrocycle series I, and the squares show those of series II. The parameters of the fit are ω_0_^I^ = 5.713 eV, and *J*^I^ = 1.472 eV (series I, excluding **C8T**), and ω_0_^II^ = 4.853 eV, and *J*^II^ = 1.051 eV (series II, excluding **C10T**). We must stress here that the parameters obtained for the fit to series I should be treated with care because of the low number of macrocycles in the series (3 elements). The difference between the ω_0_ values can be attributed to the limitation of the theory, which does not take into account the thiophene substitution pattern. For comparison, the parameters obtained for the homologous linear oligomers are ω_0_ = 5.5 eV and *J* = 1.3 eV [30]. Because of the strong sensitivity of the fit to the ω_0_ parameter, a better comparison can be performed by applying the well-established linear ω_0_ parameter to the two cyclic series, using it as a fixed value in the fitting and comparing the obtained coupling constants. These amount to *J*^I^ = 1.355 eV and *J*^II^ = 1.388 eV and reveal a slightly better electronic coupling for macrocycles of series II and therefore a reduced transition energy, which agrees with the experimentally red-shifted absorption of this series as compared to series I.

The experimentally observed linear increase of the extinction coefficients with increasing ring size (Figure 4) was also predicted by Equation 1 and Equation 2, confirming that the electronic excitation is distributed along the ring. These findings strongly corroborate the description of the electronic excitations by the model based on Frenkel excitons, despite the limitations assumed in this approach. A series of improvements of the model can be adopted in the future.

## Conclusion

Oligothiophene macrocycles exhibit interesting photophysical properties. Due to the ring geometry, the electronic transitions follow distinct selection rules and the transition dipoles are mainly arranged in the macrocycle plane. Similar to the behaviour of linear oligomers, the absorption maxima positions tended towards a fixed value with increasing size of the macrocycle. The size-dependent effects on the absorption spectra (energy and extinction coefficient) can be described in the framework of a Frenkel exciton theory. The small macrocycles show most interesting photophysical behaviour (dual fluorescence), mostly due to their strong ring strain.
